# Experiences of recruiting to a pilot trial of Cardiac Rehabilitation In patients with Bowel cancer (CRIB) with an embedded process evaluation: lessons learned to improve recruitment

**DOI:** 10.1186/s40814-015-0009-z

**Published:** 2015-04-14

**Authors:** Gill Hubbard, Anna Campbell, Zoe Davies, Julie Munro, Aileen V Ireland, Stephen Leslie, Angus JM Watson, Shaun Treweek

**Affiliations:** 1 Cancer Care Research Centre, School of Health Sciences, University of Stirling, Highland Campus, Old Perth Road, Inverness, IV2 3JH UK; 2 Faculty of Life Science, Sport and Social Sciences, Edinburgh Napier University, Sighthill Campus, Edinburgh, EH11 4BN UK; 3 Cancer Trials Unit, Tower Block 2, Room 20, UHW, Heath Park, Cardiff, CF14 4XW UK; 4 Cancer Care Research Centre, School of Health Sciences, University of Stirling, Stirling, FK9 4LA UK; 5 Cardiac Unit, Raigmore Hospital, Old Perth Road, Inverness, IV2 3UJ UK; 6 NHS Highland, Inverness, IV2 3BW UK; 7 Colorectal Surgery, Raigmore Hospital, Old Perth Road, Inverness, IV2 3UJ UK; 8 Health Services Research Unit, University of Aberdeen, 3rd Floor, Health Sciences Building, Foresterhill, Aberdeen, AB25 2ZD UK

**Keywords:** Pilot trial, Recruitment, Complex intervention, Pragmatic intervention, Cancer survivorship, Colorectal cancer

## Abstract

**Background:**

Recruitment to randomised controlled trials (RCTs) is a perennial problem. Calls have been made for trialists to make recruitment performance publicly available. This article presents our experience of recruiting to a pilot RCT of cardiac rehabilitation for patients with bowel cancer with an embedded process evaluation.

**Methods:**

Recruitment took place at three UK hospitals. Recruitment figures were based on the following: i) estimated number of patient admissions, ii) number of patients likely to meet inclusion criteria from clinician input and iii) recruitment rates in previous studies. The following recruitment procedure was used:

1. Nurse assessed patients for eligibility.
2. Patients signed a screening form indicating interest in and agreement to be approached by a researcher about the study.
3. An appointment was made at which the patient signed a consent form and was randomised to the intervention or control group.

Information about all patients considered for the study and subsequently included or excluded at each stage of the recruitment process and reasons given were recorded.

**Results:**

There were variations in the time taken to award Research Management approval to run the study at the three sites (45–359 days). Sixty-two percent of the original recruitment estimate was reached. The main reason for under-recruitment was due to over-estimation of the number of patient admissions; other reasons were i) not assessing all patients for eligibility, ii) not completing a screening form for eligible patients and iii) patients who signed a screening form being lost to the study before consenting and randomisation.

**Conclusions:**

Pilot trials should not simply aim to improve recruitment estimates but should also identify factors likely to influence recruitment performance in a future trial and inform the development of that trial’s recruitment strategies. Pilot trials are a crucial part of RCT design. Nevertheless, pilot trials are likely to be small scale, involving only a small number of sites, and contextual differences between sites are likely to impact recruitment performance in any future trial. This means that ongoing monitoring and evaluation in trials are likely to be required.

**Trial registration:**

ISRCTN63510637; UKCRN id 14092.

## Background

The randomised controlled trial (RCT) is seen as the gold standard research design when evaluating effectiveness of healthcare interventions [1]. RCTs that fail to recruit, however, may not yield reliable evidence. Underpowered studies are more likely to go unpublished or report statistically non-significant results, which increases the chances of the abandonment of interventions with potentially clinically important effects [2-4]. There are several other possible consequences arising from poor recruitment, such as increasing the cost and workload of the trial itself and ethical implications associated with recruiting patients to a trial that will ultimately fail to answer its research question [2-4].

Poor recruitment has plagued RCTs for decades [5-10], suggesting that there is no ‘quick fix’ or ‘magic solution’ to the problem [4]. A review of UK publicly funded multicentre trials (2002–2008) found that just over half of trials recruited their originally specified target sample size, just over three quarters recruited 80% of their target and just under half of trials received an extension of some kind [11]. Trial registries have improved transparency about the number of trials meeting recruitment targets but fail to capture the reasons for successful or poor recruitment, making them of limited use for understanding and addressing recruitment challenges [12]. Considerable effort has, however, been made to understand the reasons for poor or slow recruitment, and research on this subject indicates a multitude of system- and individual-level factors [13-19].

System-level factors include research governance procedures that have added to the complexity of trial procedures and as a consequence have seriously delayed recruitment [17,20,21]. Ironically, recruitment delays have arisen when protocol amendments to improve recruitment rates have been submitted to research and ethical committees for approval [16,21]. The influence of culture and context on recruitment is less well understood but will include factors such as the research infrastructure at the location where recruitment is taking place [22].

More attention has been given to individual-level factors. Given the importance of clinicians in the recruitment process, particular attention has been paid to understanding clinician-level factors [14,16,23-27]. Clinician barriers to recruiting patients include lack of time, lack of research experience and training, concerns about the impact of the trial on the doctor-patient relationship and concerns about the extra burden on patients [14,16]. Similarly, substantial effort has been made to understand patient-level factors [19,25,26,28-30]. Commonly reported patient barriers include dislike of randomisation, existing preference for a particular treatment, distrust of research and fear that involvement will negatively impact on the relationship with their doctor [17,31]. Trialists have attempted to address these barriers to recruitment, although systematic reviews of studies evaluating interventions to increase recruitment to RCTs suggest that few interventions have a solid evidence base [2,4,32-34].

In order to contribute towards addressing the problem of recruitment, calls have been made for trialists to publish their experiences of recruitment and to make data regarding recruitment performance publicly available [1,20,35]. This article presents our experience of recruiting to a pilot RCT of cardiac rehabilitation for patients with bowel cancer [36]. Key aims of the pilot trial were to determine eligibility, consent, recruitment and retention rates in preparation for a future large-scale effectiveness RCT. Our findings may be of use to other trialists addressing recruitment difficulties in similar trials.

## Methods

### Study design

Data were drawn from the Cardiac Rehabilitation In Bowel cancer patients (CRIB) pilot trial, the design of which is described elsewhere [36]. Briefly, CRIB is a two-arm pilot RCT to assess the effectiveness of cardiac rehabilitation on bowel cancer patients’ level of physical activity, quality of life, fatigue, anxiety and depression compared to patients in the control arm receiving the ‘Staying healthy after bowel cancer’ booklet produced by Bowel Cancer UK, which includes a section on ‘staying fit’ [37]. This paper reports the analysis of recruitment data for this pilot trial.

### Recruitment setting

Recruitment took place at three UK hospitals where patients with bowel cancer were admitted for surgery and where cardiac rehabilitation is also available on the site. Site 1 included patients who lived in remote and rural areas where access to services such as cardiac rehabilitation may be an issue [38,39]. The other two sites served an urban population.

### Participants

#### Inclusion

1. Adults who have been diagnosed with primary colorectal cancer and are in the recovery period post-surgery.
2. Patients receiving adjunctive chemotherapy/radiotherapy are included. Patients must wait 48 h post-chemotherapy before taking part in the intervention.

#### Exclusion

1. Patients with advanced disease.
2. Patients who fail clinical/risk assessment for rehabilitation and are deemed unsafe to participate in exercise classes. (According to recent guidelines, those with severe anaemia should delay exercise and patients with compromised immune function should avoid public gyms and exercise classes [40]).
3. Patients with severe cognitive impairment and who therefore are unable to give informed consent to participate in the study, or are unable to communicate in English as this is the language used in the delivery of cardiac rehabilitation.

### Approvals

An application for National Health Service (NHS) ethics approval was submitted using the electronic Integrated Research Application System (IRAS) [41]. Applications for NHS Research Management approval, an additional approval required in the UK for research involving NHS patients, staff or premises, were made to the Research and Development office in each of the three Health Boards conducting the study.

### Sample size estimation

The aim of our pilot was not to provide a definitive estimate of treatment effect, so we did not have a formal sample size calculation. Rather, the aim was to provide robust estimates of the likely rates of recruitment and retention and to yield estimates of the variability of the primary and secondary outcomes to inform power calculations for a future large-scale effectiveness trial. Our recruitment calculation for the pilot trial was based on three factors:

- Estimated number of patients admitted for surgery (based on previous annual admissions)
- Number of patients likely to meet inclusion criteria based on clinician input
- Recruitment rates in previous similar studies (e.g. trials of physical activity interventions for people with cancer).

Based on information provided by the local NHS principal investigators of the number of patient admissions in the previous year (2012), we expected 250 patients, in total, to be admitted for surgery across the three sites over a 6-month period. Cancer clinicians involved in the study estimated that approximately one third (*n* = 83) would be ineligible, and based on recruitment to an RCT of physical activity with patients with cancer in Scotland (27% recruitment rate) [42] and a trial involving patients with colorectal cancer within 3 months of completing surgery conducted in Canada (35% recruitment rate) [43], we estimated that just over a third of eligible patients would consent (*n* = 66) to take part. Thus, for the pilot RCT, we expected to recruit around 66 patients (40% of eligible patients). We estimated that sites 2 and 3 would recruit 26 patients, respectively, and that site 1 would recruit 14 patients, as this site admitted fewer patients for surgery compared with the other two sites. These are the recruitment, eligibility and consent rates that we had in the study protocol that was approved by the NHS research and ethics committee.

### Recruitment and consent

Recruitment took place over 6 months, from 1 January to 31 July 2014. The recruitment process had several stages, and at each stage, patients could withdraw. At sites 1 and 2, the following procedure was followed:

1. A colorectal clinical nurse specialist assessed patients admitted for surgery for eligibility using medical notes and knowledge about the patients. The nurse, using free text, recorded reasons for ineligibility.
2. Patients who were eligible were given an information sheet. This took place either pre-or post-surgery on the ward and was carried out by a clinical nurse specialist. Screening forms, which were required for all eligible patients, provided clinical (e.g. date of surgery, adjuvant treatments) and demographic (e.g. age, gender) information. Patients signed this form on the ward if they were interested in participating and willing to be approached by a researcher about the study at a later date. The form was also signed by those who did not wish to participate but who were willing to have information about them retained for the purposes of the study (i.e. to evaluate if recruited patients were representative of eligible patients). If a screening form was not completed, then the patient was lost to the study.
3. A researcher contacted each patient who signed a screening form indicating willingness to participate in the study by telephone, and they were given further information about the study. An appointment was made at which the patient signed a consent form and was randomised to the intervention or control group.

Recruitment at site 3 was slightly different because a research nurse carried out all three stages of the recruitment process as opposed to the combined effort of nurses and a researcher. Research nurses are nurses employed by hospitals in the UK to recruit to RCTs and can be working on a large number of trials simultaneously.

### Data collection and analysis

Recruitment performance was closely monitored and discussed at a monthly meeting by the three researchers (AI, ZD, JM) in each site and the PI (GH). The screening form included the following information: date of surgery, cancer diagnosis (Dukes or AJCC-TNM staging), type of surgery, method of surgery, type of stoma (if applicable), type of adjuvant therapy (if any) and inclusion and exclusion criteria. These data were entered into the OpenClinica (https://www.openclinica.com) data management system developed by Tayside Clinical Trials Unit for the study. The database also included the following fields: if patient was given information sheet; patient permission OR reason for not taking part. Free text was used by nurses, research nurses and researchers involved in the recruitment process to describe reasons why eligible patients did not wish to participate, which, for the purpose of analysis, were sorted into one of seven categories:

- No longer eligible
- Distance/travel
- Stated that they are currently exercising and fit
- Clinical (e.g. poor recovery from surgery, receiving adjuvant therapy, co-morbidity)
- Too much of a commitment
- Not/no longer interested
- No reason given.

Thus, information about all patients considered for the study and subsequently included or excluded at each stage of the recruitment process and reasons given were recorded. Aggregated descriptive presentations of recruitment data for all sites and for each of the three sites were made and reported below.

## Results

### Approvals

An application for NHS ethical approval was submitted 12 months prior to the planned recruitment start date (January 2014). The submission was made on 20 January 2013, received by the NHS ethical committee on 25 January and reviewed by the committee at a meeting on 14 February 2013 (REC reference 13/NS/0004; IRAS project ID 121757). The committee requested further information and submission of revised documentation. This request was submitted to the Chair of the ethical committee on 21 February 2013, and a favourable ethical opinion was given on 22 February 2013.

NHS Research Management approval was sought from each of the three sites at the same time as NHS ethics committee approval was sought, which is normal practice in the UK. There were substantial variations in the time taken to award Research Management approval to run the study at the three sites:

- Site 1: 05 March 2013 (45 days)
- Site 2: 17 December 2013 (331 days)
- Site 3: 14 January 2014 (359 days).

There are three main reasons to account for this variation. Firstly, the request for Research Management approval was not directed to the correct person in the Research Office at site 3 and was not dealt with until the correct person got the application for approval several months after the application was submitted. Chasing an application at a distance by email and telephone proved difficult and caused delays. Secondly, the information requested by research managers across the sites differed, although all three sites were approving the same piece of research. For instance, the research manager at site 2 insisted that the contract between the universities employing the co-applicants on the grant be signed before giving approval. This was not required at the other two sites. Third, the principal investigator for the study was based at site 1 and had local contacts in the NHS Research Office, which may have contributed towards quickly obtaining approval.

### Original estimated and actual recruitment rates

**Figure 1:**
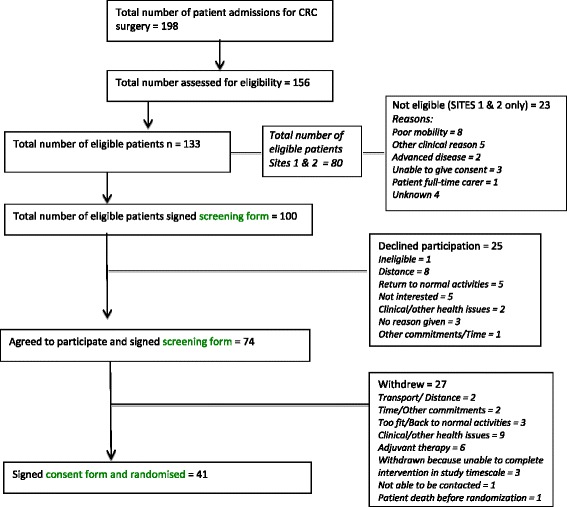
Recruitment flowchart.

Figure 1 shows patient flow throughout the study.

In total, 41 patients were recruited to the study, which is 62% of our original estimate of 66 recruited patients. Table 1 shows the difference between estimated and actual patient admissions, eligibility and consent rates across all three sites. Figure 1 shows graphically the difference between estimated and actual patient admissions at each stage of the recruitment process.

**Table 1:** Revised estimated and actual admission, eligibility and consent rates in each site

	Site 1		Site 2		Site 3		All sites		
	Estimated	Actual	Estimated	Actual	Estimated	Actual	Original estimate	Revised estimated	Actual
Admissions	74	58	134	58	125	82	250	333	198
Eligible (% of admissions)	49 (66%)	40 (69%)	88 (66%)	40 (69%)	82 (66%)	53 (65%)	165 (66%)	219 (66%)	133 (67%)
Randomised (% of eligible patients)	20 (40%)	13 (32%)	35 (40%)	18 (45%)	33 (40%)	10 (19%)	66 (40%)	88 (40%)	41 (31%)

The number of actual surgical admissions was lower than expected (198 vs 250). We correctly estimated the proportion of patient admissions that would be eligible (i.e. approximately two thirds); 133 out of 198 actual patient admissions were judged as eligible for the study (67%). However, because we had initially over-estimated the number of patient admissions, there was a difference of 20% between expected and actual number of eligible patients. We estimated that 66 patients would consent to study participation and be randomised. Seventy-four patients signed a screening form indicating that they were interested in participating (see Table 2), but only 41 of these patients were actually randomised into the study. Thirty-one percent as opposed to an estimated 40% of eligible patients were randomised.

**Table 2:** Recruitment activity for each site

	Site 1	Site 2	Site 3	All sites
Admissions (1 January to 31 July 2014)	58	58	82	198
Stage 1: Assessing patients for eligibility				
Assessed for eligibility Proportion of patient admissions (*n* = 58, 58, 82)	50 86%	53 91%	53 65%	156 79%
Number of eligible patients Proportion of patient admissions (*n* = 50, 53, 53)	40 67%	40 67%	53 65%	133 67%
Stage 2: Screening forms				
Screening forms for eligible patients Proportion of eligible patients (*n* = 40, 40, 53)	32 80%	31 78%	37 70%	100 75%
Consented to be approached by researcher Proportion of screening forms (*n* = 32, 31, 37)	23 72%	23 74%	28 76%	74 74%
Declined to participate Proportion of screening forms (*n* = 32, 31, 37)	9 28%	8 26%	8 21%	25 25%
Ineligible Proportion of screening forms (*n* = 32, 31, 37)	0 0%	0 0%	1 3%	1 1%
Stage 3: Randomisation				
Randomised Proportion consenting to be approached (*n* = 23, 23, 28)	13 56%	18 78%	10 37%	41 55%
Withdrew before consent/randomisation Proportion consenting to be approached (*n* = 23, 23, 28)	8 35%	5 22%	14 50%	27 36%
Withdrawn because would not complete cardiac rehabilitation within timescale Proportion consenting to be approached (*n* = 23, 23, 28)	0 0%	0 0%	3 11%	3 4%
Not able to be contacted successfully Proportion consenting to be approached (*n* = 23, 23, 28)	2 9%	0 0%	1 3%	3 4%

### Revised estimated and actual recruitment rates

**Figure 2:**
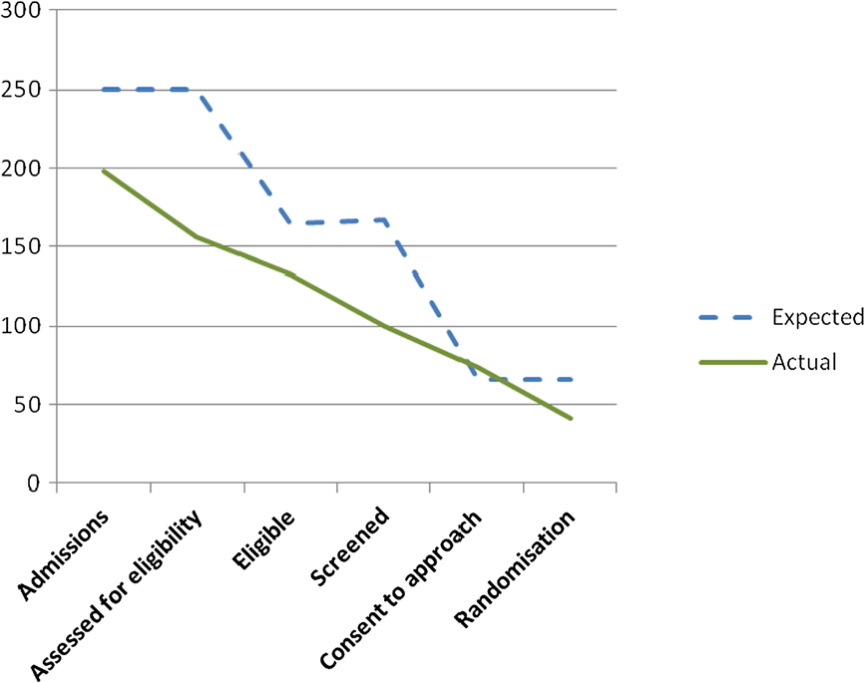
Estimated versus actual recruitment rate across all three sites.

**Figure 3:**
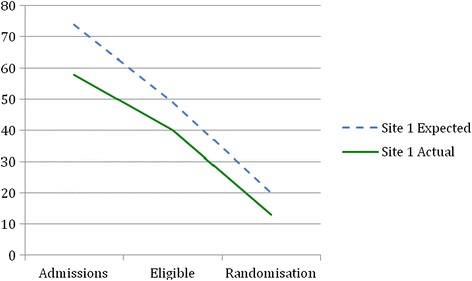
Site 1 revised estimated and actual recruitment rate.

**Figure 4:**
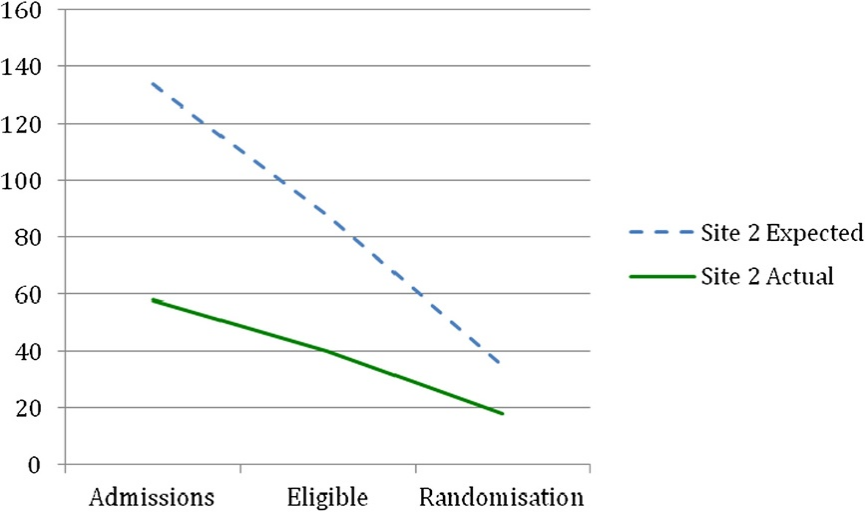
Site 2 revised estimated and actual recruitment rate.

**Figure 5:**
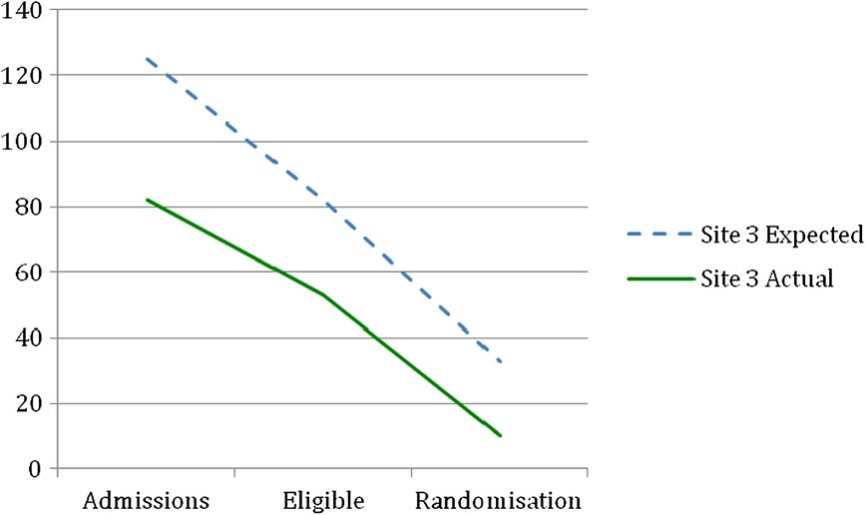
Site 3 revised estimated and actual recruitment rate.

Before recruitment actually started, recruitment estimates were revised by nurses involved in recruitment in each site using records of the number of patients admitted for surgery in the previous year (2012). The reason for requesting a revised figure was to obtain an estimate from those clinicians who would be actually involved in recruitment, using their records. Table 1 shows the difference between revised estimated and actual patient admissions, eligibility and consent rates in each site. Figures 2, 3, 4 and 5 show graphically the difference between revised estimated and actual patient admissions at each stage of the recruitment process in each site.

Table 1 and Figures 2, 3, 4 and 5 show that all sites over-estimated the number of patient admissions. The main reason why we did not meet our recruitment target was due to over-estimation of the number of patient admissions in all sites. However, all sites achieved the estimated percentage of eligible patients, i.e. 66%. We had estimated that 40% of all eligible patients would be randomised; site 1 randomised 32% and site 3 randomised 19% of eligible patients. Site 2, in contrast, randomised 45% of eligible patients. Why sites did not manage to meet their estimated recruitment target is explained in the following sections.

### Stage 1: Assessing patients for eligibility

Table 2 shows that the research nurse in site 3 assessed 65% of patients for eligibility, whereas the clinical nurse specialists at the other two sites reached more patients, assessing 86% and 91% of all patient admissions, respectively. Although for the purposes of trial we wanted every patient formally assessed and the reasons for ineligibility reported, the research nurse at site 3 said during one of the monthly research team meetings that she had deliberately only assessed those patients whom she knew were most likely to be eligible and had avoided those she was almost certain would not meet eligibility criteria. This strategy meant that more patients seemed to be lost to the study at this stage of the recruitment process at site 3 than at sites 1 and 2. How much of a difference to recruitment formally assessing more patients for eligibility at site 3 would have made is debatable as the proportion of admissions that were eligible was almost identical across the three sites (67%, 67% and 65%, respectively).

Nurses at sites 1 and 2 recorded the reasons why patients were considered ineligible (*N.B.* this information was not collected at site 3). Table 3 shows that the main reason why patients were considered to be ineligible was poor mobility. The table shows that over half (57%) of patients were excluded because of poor mobility or other clinical reasons. When these reasons are mapped to the exclusion criteria listed in the ‘Participants’ section, it is clear that the main reason for ineligibility is criterion 2, i.e. ‘Patients who fail clinical/risk assessment for rehabilitation and are deemed unsafe to participate in exercise classes’.

**Table 3:** Reasons for ineligibility (sites 1 and 2)

Reason given by nurse	Number of patients	Exclusion criteria (1–3)^a^
Poor mobility	8 (35%)	2
Other clinical reason	5 (22%)	2
Advanced disease	2 (9%)	1
Unable to provide consent	3 (13%)	3
Patient is a full-time carer	1 (4%)	N/A
Unknown	4 (17%)	N/A

Percentages are the proportion of ineligible patients at sites 1 and 2, *n* = 23.

*N*/*A* not applicable.

^a^See ‘Participants’ section.

### Stage 2: Screening forms

Table 2 above shows that screening forms were completed for 75% of all eligible patients across the three sites. Table 4 shows the reasons why screening forms were not completed at the three sites.

**Table 4:** Reasons why a screening form was not completed

Reason	Site 1	Site 2	Site 3
Early discharge or moved wards	4 (10%)	5 (12%)	0 (0%)
Refused to have information kept	4 (10%)	4 (10%)	0 (0%)
Patient could not be consented in time to start intervention	0 (0%)	0 (0%)	16 (30%)

Percentages are the proportion of eligible patients (site 1 *n* = 40, site 2 *n* = 40, site 3 *n* = 53).

At site 3, 30% of eligible patients did not complete a screening form because the research nurse judged that it would be unlikely that the patient would be able to start the intervention (i.e. cardiac rehabilitation) within the allocated time period of the study. The protocol states that patients could attend cardiac rehabilitation 6 weeks post-laparoscopic and 8 weeks post-open surgery. Patients could attend cardiac rehabilitation while they were receiving adjuvant therapy, but at site 3, the research nurse and/or patient did not think that they would be able to simultaneously manage adjuvant therapy and exercise. Completing a screening form for these patients was therefore perceived as an inappropriate use of the research nurse and patient time because the patient was unlikely to be entered into the study.

### Stage 3: Randomisation

Table 2 shows that 25% of eligible patients completing a screening form did not agree to participate across the three sites. The table also shows that during stage 3, 35%, 22% and 50% of patients who initially had indicated on the screening form that they were interested in participating did not agree to participate in the study at sites 1, 2 and 3, respectively.

Table 5 shows the reasons why patients did not agree to participate at stages 2 and 3. The most common reasons (40%) fell into the clinical category, which included poor recovery from surgery, co-morbidity or receiving adjuvant therapy. At sites 2 and 3, at least half of eligible patients cited clinical reasons for non-participation compared to just under a fifth at site 1. Site 1 included patients living in remote and rural areas.

**Table 5:** Reasons for declining to participate

Reason	Site 1	Site 2	Site 3	All sites
No longer eligible	2 (11.5%)	-	2 (9%)	4 (7%)
Distance/travel barriers	7 (41%)	-	2 (9%)	9 (17%)
Perceived as already exercising and fit	3 (18%)	2 (15%)	1 (4%)	6 (12%)
Clinical, e.g. poor recovery from surgery, receiving adjuvant therapy, co-morbidity	3 (18%)	7 (54%)	11 (50%)	21 (40%)
Too much of a commitment	-	-	3 (14%)	3 (6%)
Not/no longer interested	2 (11.5%)	4 (31%)	-	6 (12%)
No reason given	-	-	3 (14%)	3 (6%)

Percentages are of the proportion of patient refusals (site 1 *n* = 17, site 2 *n* = 13, site 3 *n* = 22).

## Discussion

Recruitment is frequently the most difficult task in conducting an RCT [35]. Calls for public reporting of recruitment performance for each site within a trial, not just overall recruitment [35], will improve transparency but will not necessarily improve recruitment. Understanding barriers and developing interventions to improve recruitment are also required.

System-level barriers to recruitment include research, ethical and management approvals, which have been found to have a detrimental impact on recruitment in trials [16,17,20]. Although we did not encounter problems in obtaining NHS ethical committee approval, we did experience problems with NHS Research Management approval at two of the three sites. We found inconsistencies between sites for obtaining Research Management approvals. For example, one research manager insisted that all contracts between universities where grant holders were employed must be signed before NHS Research and Management approval could be granted. A single Research and Management approval system and guidance for researchers and research managers may contribute towards easing the process. In addition, face-to-face communication between the research team and the NHS Research and Development office may also help avoid delays in obtaining NHS Research and Management approval.

Many reasons for low levels of recruitment have been cited in the literature, including fewer eligible patients than expected and a smaller percentage of patients actually agreeing to participate than originally estimated [15,44]. Recruitment estimates can be used to monitor recruitment performance, and therefore, it is helpful to get estimates as accurate as possible. This is why it is sometimes helpful to revise estimates if more robust data to inform estimates are obtainable. Our experience suggests that obtaining the number of patient admissions is not as straightforward as it seems. For instance, our original estimate that we used in the protocol differed from our revised estimate. More importantly, the actual number of patient admissions during the recruitment period differed from both of these estimates. Trialists can make recruitment estimates based on the literature, routine administrative data and experience; by conducting feasibility and pilot work, a trialist hopes to improve these estimates. It is important that this assumption of improved estimates is a good one because doing feasibility and pilot studies requires resources. Dickson and colleagues, for instance, conducted a pilot trial in two sites but in the main trial still encountered recruitment problems due to fewer eligible women presenting to participating clinics than predicted [20]. McDonald and colleagues found that trials having pilot phases, in many cases, changed their recruitment strategies as a result of them. Despite this, they found no difference in recruitment success between trials having a pilot phase and those that did not [8]. If preliminary studies are only used to improve estimates and little effort is expended in understanding barriers and facilitators to recruitment, then any future trial may nevertheless encounter recruitment difficulties.

A pilot trial is particularly useful in pinpointing where in the recruitment pathway barriers to participation are most likely to occur. Our pilot trial, for instance, suggests that stage 3 (consenting and randomisation) was where most participants were lost to the study. It seems reasonable therefore to focus on the part of the pathway where most patients are lost to the study. However, recruitment is best conceived as a whole system with inter-related discrete stages and processes. Any change in an earlier part of the system will have a knock-on effect on other parts. It may be more productive, therefore, to focus attention on earlier stages, i.e. stages 1 (screening for eligibility) and 2 (participant agreeing to be involved and contacted by a researcher), in order to improve recruitment in stage 3 (consenting and randomisation). Understanding recruitment as a process rather than a singular event was highlighted at a recent trial recruitment workshop [45].

Studies have consistently shown that clinicians have a significant impact on recruitment performance [14,17,23-26]. Training of clinical recruiters may improve recruitment rates [46]. The literature does not refer to behaviour models or theories to understand or explain recruitment performance, preferring instead to highlight factors such as lack of time [24,47]. Behavioural theories may contribute towards understanding recruitment performance. In social cognitive theory, for instance, ‘outcome expectations’ reflect individuals’ beliefs about what consequences are most likely to ensue if particular behaviours are performed [48]. Applied to recruitment, the theory suggests that clinicians may not adhere to recruitment protocols if they believe that it will not actually make a difference to the recruitment rate. Our pilot trial shows that the research nurse at site 3 only assessed those patients who she knew were likely to be eligible and did not formally assess those who she believed would not be eligible, which suggests that she did not implement research procedures that she believed would not affect the overall recruitment rate of the study. Additionally, she did not complete a screening form for those patients who would be having adjuvant therapy and therefore unable to attend cardiac rehabilitation. The general point we are making is that recruitment is not simply a practical venture necessitating practical solutions to improve recruitment performance (e.g. addressing lack of time) but requires understanding and addressing the behaviour (e.g. assessing outcomes expectancies) of both staff and patients.

Few studies have examined differences in recruitment by health profession [17]. We found that clinical practice nurses assessed more patients for eligibility than a research nurse did. Nurses possibly know patients better than research nurses because they are providing care and it may be that nurses can assess patients for eligibility very quickly, without recourse to reading medical notes or speaking with the patient. Needless to say, it is difficult to draw a definitive conclusion about differences in recruitment by profession from such a small study, and further research examining recruitment performance by profession is required.

The literature has highlighted patient barriers to recruitment [31,17]. An important issue to emerge in our study was the need to look at issues to do with recruitment at a site-specific level because barriers to recruitment vary across sites. Conducting a pilot trial in a small number of sites with a range of features considered relevant to recruitment may be useful in pre-empting common and unique patient barriers within different contexts. The pilot trial, for instance, suggests that only remote and rural sites are likely to experience distance/travel factors as a barrier to recruitment but that all sites are likely to experience poor recovery and ongoing treatment as patient barriers to participation. The importance of context for understanding the conduct and outcomes of trials has been recognised in other research [49,50]. Ongoing monitoring and evaluation could be conducted in a small number of sites representative of other sites with shared characteristics on the assumption that recruitment barriers and therefore solutions will be applicable to those sites with common characteristics and similar contexts. This may be a more cost-effective solution than ongoing evaluation and monitoring in every single site in a larger trial.

There are common barriers to recruitment [13-19], and awareness of these should help trialists to develop strategies to address typical barriers. Nevertheless, reasons for poor recruitment, and thereby strategies for improving recruitment, will vary from one trial to the next. One trial, for instance, reported poor clinician ‘buy in’ as a factor impeding recruitment [51], whereas this was not an issue that we encountered in our pilot trial. Another trial cited protocol issues as the main reason for poor participation [52], whereas our pilot trial identified clinical (e.g. poor recovery from surgery, receiving adjuvant therapy, co-morbidity) and distance/travel issues as the main reasons for refusal. This is why it is important to continuously and closely monitor recruitment and use qualitative methods to identify and then rectify problems through the use of tailored interventions [2,4,12,53].

### Strengths and limitations

This pilot trial shows system- and individual-level factors impacting recruitment of patients with bowel cancer to a pilot RCT of cardiac rehabilitation. Caution is required when interpreting these findings because they are drawn from a small pilot trial involving only three sites. There is inevitably a limit to the generalisability of these findings beyond this particular population, intervention and study design. Furthermore, even equivalent population and intervention trials are unlikely to face identical barriers because of the influence of contextual factors [22]. In other words, generalisability will always be limited. Nevertheless, there may be common methods that trialists can adopt to improve recruitment, including the careful use of pilot trials and ongoing monitoring and evaluation of recruitment performance.

## Conclusions

Pilot trials should not simply aim to improve recruitment estimates but should also identify factors likely to influence recruitment performance in a future large-scale trial and inform the development of that trial’s recruitment strategies. Pilot trials are a crucial part of RCT design and should be fully supported and funded. Nevertheless, pilot trials are likely to be small scale, involving only a small number of sites, and contextual differences between sites are likely to impact recruitment performance in any future trial. This means that factors impacting recruitment ought to be examined at a site-specific level. This means that ongoing monitoring and evaluation in effectiveness trials are likely to be required.

## Abbreviations

CRIB: Cardiac rehabilitation in bowel cancer study
RCT: Randomised controlled trial
UK: United Kingdom

## References

[1] Odgaard-Jensen J, Vist GE, Timmer A, Kunz R, Akl EA, Schünemann H (2011). Randomisation to protect against selection bias in healthcare trials. Cochrane Database Syst Rev.

[2] Fletcher B, Gheorghe A, Moore D, Wilson S, Damery S (2012). Improving the recruitment activity of clinicians in randomized controlled trials: a systematic review. BMJ Open.

[3] Kaur G, Smyth RL, Williamson P (2012). Developing a survey of barriers and facilitators to recruitment in randomized controlled trials. Trials.

[4] Treweek S, Lockhart P, Pitkethly M, Cook JA, Kjeldstrøm M, Johansen M (2013). Methods to improve recruitment to randomized controlled trials: Cochrane systematic review and meta-analysis. BMJ Open.

[5] Charleson ME, Horwitz R (1984). Applying results of randomised controlled trials to clinical practice: impact of losses before randomisation. Br Med J (Clin Res Ed).

[6] Easterbrook PJ, Matthews DR (1992). Fate of research studies. J R Soc Med.

[7] Foy R, Parry J, Duggan A (2003). How evidence based are recruitment strategies to randomised controlled trials in primary care? Experience from seven studies. Fam Pract.

[8] McDonald AM, Knight RC, Campbell MK, Entwistle VA, Grant AM, Cook JA (2006). What influences recruitment to randomised controlled trials? A review of trials funded by two UK funding agencies. Trials.

[9] Bower P, Wilson S, Mathers N (2007). Short report: how often do UK primary care trials face recruitment delays?. Fam Pract.

[10] Toerien M, Brookes ST, Metcalfe C (2009). A review of reporting of participant recruitment and retention in RCTs in six major journals. Trials.

[11] Sully BGO, Julious SA, Nicholl J (2013). A reinvestigation of recruitment to randomised, controlled, multicenter trials: a review of trials funded by two UK funding agencies. Trials.

[12] Mitchell AP, Hirsch BR, Abernethy AP (2014). Lack of timely accrual information in oncology clinical trials: a cross-sectional analysis. Trials.

[13] Fayter D, McDaid C, Eastwood A (2007). A systematic review highlights threats to validity in studies of barriers to cancer trial participation. J Clin Epidemiol.

[14] Prescott RJ, Counsell CE, Gillespie WJ, Grant AM, Russell IT, Kiauka S (1999). Factors that limit the quality, number and progress of randomised controlled trials. Health Technol Assess.

[15] Campbell MK, Snowdon C, Francis D, Elbourne D, McDonald AM, Knight R (2007). Recruitment to randomised trials: strategies for trial enrolment and participation study. The STEPS study. Health Technol Assess.

[16] van Staa T-P, Dyson L, McCann G, Padmanabhan S, Belatri R, Goldacre B (2014). The opportunities and challenges of pragmatic point-of-care randomised trials using routinely collected electronic records: evaluations of two exemplar trials. Health Technol Assess.

[17] Ellis PM (2000). Attitudes towards and participation in randomised clinical trials in oncology: a review of the literature. Ann Oncol.

[18] Cox K, McGarry J (2003). Why patients don’t take part in cancer clinical trials: an overview of the literature. Eur J Cancer Care.

[19] Martin S, Ou FS, Newby LK, Sutton V, Adams P, Felker GM (2013). Patient- and trial-specific barriers to participation in cardiovascular randomized clinical trials. J Am Coll Cardiol.

[20] Dickson S, Logan J, Hagen S, Stark D, Glazener C, McDonald AM (2013). Reflecting on the methodological challenges of recruiting to a United Kingdom-wide, multi-centre, randomised controlled trial in gynaecology outpatient settings. Trials.

[21] Treweek S, Wilkie E, Craigie AM, Caswell S, Thompson J, Steele RJC (2013). Meeting the challenges of recruitment to multicentre, community-based, lifestyle-change trials: a case study of the BeWEL trial. Trials.

[22] Bates P (2014). Context is everything.

[23] Rendell JM, Merritt RK, Geddes J (2007). Incentives and disincentives to participation by clinicians in randomised controlled trials. Cochrane Database Syst Rev.

[24] Taylor KM, Margolese RG, Soskolne CL (1984). Physicians’ reasons for not entering eligible patients in a randomized clinical trial of surgery for breast cancer. N Engl J Med.

[25] Ross S, Grant A, Counsell C, Gillespie W, Russell I, Prescott R (1999). Barriers to participation in randomised controlled trials: a systematic review. J Clin Epidemiol.

[26] Lovato LC, Hill K, Hertert S, Hunninghake DB, Probstfield JL (1997). Recruitment for controlled clinical trials: literature summary and annotated bibliography. Control Clin Trials.

[27] Donovan JL, Parmasivan S, de Salis I, Torrien M (2014). Clear obstacles and hidden challenges: understanding recruiter perspectives in six pragmatic randomised controlled trials. Trials.

[28] Sharp L, Cotton SC, Alexander L, Williams E, Gray NM, Reid JM (2006). Reasons for participation and non-participation in a randomized controlled trial: postal questionnaire surveys of women eligible for TOMBOLA (Trial Of Management of Borderline and Other Low-Grade Abnormal smears). Clin Trials.

[29] Smyth RMD, Duley L, Jacoby A, Elbourne D (2009). Women’s experiences of participating in the Magpie Trial: a postal survey in the United Kingdom. Birth.

[30] Virani S, Burke L, Remick SC, Abraham J (2011). Barriers to recruitment of rural patients in cancer clinical trials. J Oncol Pract.

[31] Mills EJ, Seely D, Rachlis B, Griffith L, Wu P, Wilson K (2006). Barriers to participation in clinical trials of cancer: a meta-analysis and systematic review of patient-reported factors. Lancet Oncol.

[32] Watson JM, Torgerson DJ (2006). Increasing recruitment to randomized trials: a review of randomised controlled trials. BMC Med Res Methodol.

[33] Mapstone J, Elbourne D, Roberts I (2007). Strategies to improve recruitment to research studies. Cochrane Database Syst Rev.

[34] Caldwell PHY, Hamilton S, Tan A, Craig JC (2010). Strategies for increasing recruitment to randomised controlled trials: systematic review. PLoS Med.

[35] Dal-Re’ R, Moher D, Gluud C, Treweek S, Demotes-Mainard J, Carné X (2011). Disclosure of investigators’ recruitment performance in multicenter clinical trials: a further step for research transparency. PLoS Med.

[36] Munro J, Adams R, Campbell A, Campbell S, Donaldson C, Godwin J (2014). CRIB—the use of cardiac rehabilitation services to aid the recovery of patients with bowel cancer: a pilot randomised controlled trial (RCT) with embedded feasibility study. BMJ Open.

[37] Bowel Cancer UK. ‘Staying Healthy after Bowel Cancer’ leaflet. http://www.bowelcanceruk.org.uk/media/75220/staying_healthy.pdf (accessed 20 Jan 2014).

[38] Befort C, Austin H, Klemp J (2011). Weight control needs and experiences among rural breast cancer survivors. Psychooncology.

[39] Rogers LQ, Markwell SJ, Courneya KS, McAuley E, Verhulst S (2009). Exercise preference patterns, resources, and environment among rural breast cancer survivors. J Rural Health.

[40] Rock C, Doyle C, Demark-Wahnefried W, Meyerhardt J, Courneya KS, Schwartz AL (2012). Nutrition and physical activity guidelines for cancer survivors. CA Cancer J Clin.

[41] IRAS Integrated Research and Application System. https://www.myresearchproject.org.uk. Accessed August 2012

[42] Mutrie N, Campbell A, Whyte F, McConnachie A, Emslie C, Lee L (2007). Benefits of supervised group exercise intervention in cancer patients undergoing chemotherapy: randomised controlled trial. BMJ.

[43] Courneya K, Friedenreich C, Quinney H, Fields A, Jones L, Fairey A (2003). A randomized trial of exercise and quality of life in colorectal cancer survivors. Eur J Cancer Care.

[44] Eborall HC, Stewart MC, Cunningham-Burley S, Price JF, Fowkes FG (2011). Accrual and drop out in a primary prevention randomised controlled trial: qualitative study. Trials.

[45] Patterson S, Mairs H, Borschmann R (2011). Successful recruitment to trials: a phased approach to opening gates and building bridges. BMC Med Res Methodol.

[46] Campbell A, Whyte F, Mutrie N (2005). Training of clinical recruiters to improve recruitment to an exercise intervention during breast cancer treatment. Clin Eff Nurs.

[47] Taylor KM, Feldstein ML, Skeel RT, Pandya KJ, Ng P, Carbone PP (1994). Fundamental dilemmas of the randomized clinical trial process: results of a survey of the 1,737 Eastern Cooperative Oncology Group investigators. J Clin Oncol.

[48] Bandura A (2001). Social cognitive theory: an agentic perspective. Annu Rev Psychol.

[49] Wells M, Willliams B, Treweek S, Coyle J, Taylor J (2012). Intervention description is not enough: evidence from an in-depth multiple case study on the untold role and impact of context in randomised controlled trials of seven complex interventions. Trials.

[50] Hawe P, Shiell A, Riley T (2009). Theorising interventions as events in systems. Am J Community Psychol.

[51] Peters-Lawrence MH, Bell MC, Hsu LL, Osunkwo I, Seaman P, Blackwood M (2012). Clinical trial implementation and recruitment: lessons learned from the early closure of a randomized clinical trial. Contemp Clin Trials.

[52] Brintnall-Karabelas J, Sung S, Cadman ME, Squires C, Whorton K, Pao M (2011). Improving recruitment in clinical trials: why eligible participants decline. J Empir Res Hum Res Ethics.

[53] Kanarek NF, Kanarek MS, Olatoye D, Carducci MA (2012). Removing barriers to participation in clinical trials, a conceptual framework and retrospective chart review study. Trials.

